# Childhood Passive Smoking Exposure and Age at Menarche in Chinese Women Who Had Never Smoked: The Guangzhou Biobank Cohort Study

**DOI:** 10.1371/journal.pone.0130429

**Published:** 2015-07-17

**Authors:** Shanshan Yang, Yali Jin, Yao He, Chaoqiang Jiang, Kar Keung Cheng, Weisen Zhang, Tai Hing Lam

**Affiliations:** 1 Institute of Geriatrics, Chinese PLA General Hospital, Beijing, China; 2 Guangzhou Number 12 People’s Hospital, Guangzhou, China; 3 Public Health, Epidemiology and Biostatistics, University of Birmingham, Birmingham, United Kingdom; 4 School of Public Health, the University of Hong Kong, Hong Kong, China; 5 Jinan Military Area CDC, Jinan, Shandong, 250014, China; 6 Beijing Key Laboratory of Aging and Geriatrics, Chinese PLA General Hospital, Beijing, China; 7 State Key Laboratory of Kidney Disease, Chinese PLA General Hospital, Beijing, China; The University of Auckland, NEW ZEALAND

## Abstract

**Objective:**

We examined the associations between childhood passive smoking exposure and age at menarche in women who had never smoked in southern China.

**Methods:**

Among 30,518 participants in Guangzhou Biobank Cohort Study (GBCS) from 2003-2008, 20,061 women who had never smoked and had complete outcome data were included. Childhood passive smoking exposure was defined as living with 1 or more smokers in the same household during childhood. Data on the number of smokers in the household and frequency of exposure (density and frequency) were also obtained. Age at menarche was measured as a continuous variable.

**Results:**

11,379 (56.7%) participants were exposed to passive smoking during childhood. Compared to those with no passive smoking exposure during childhood, those with exposure ≥5 days/week had menarche 0.19 year (95% confidence interval (CI): 0.13-0.25) earlier on average. Those exposed to more than two smokers had menarche 0.38 year earlier (95% CI: 0.29-0.47). Childhood exposure was associated with early age at menarche (≤13 vs. >13 years), with an adjusted odds ratio of 1.34 (95% CI: 1.21-1.48) for high density, and 1.17 (95% CI: 1.09-1.26) for high frequency of exposure.

**Conclusion:**

Childhood passive smoking exposure was associated with earlier age at menarche, with a dose-response relationship in Chinese women who had never smoked. If causal, the results support the promotion of smoking cessation in families with children, particularly young girls.

## Introduction

Early menarche has been associated with fertility [1], ovarian function [2], endometrial cancer [3], breast cancer [4,5], cardiovascular diseases [6], type 2 diabetes [7,8], osteoporosis [9], high blood pressure [10], and mortality [6,11]. A downward trend in the age at menarche has been observed in Asia [12], following that in developed countries, which began in the late 19th century [13,14]. In China, the age at menarche decreased from 16.1 to 14.3 years from 1930 to 1970 [15].

A strong correlation between a mother's and her daughter's age at menarche has been reported in recent studies, suggesting that genetic and family factors influence the age at menarche [16,17]. Environmental and lifestyle factors (such as father's income index, urban birthplace, birth length [18], and body size [19]) also have impacts on the age at menarche. In addition, an association between maternal smoking during pregnancy and an earlier onset of menarche [20] was reported. A previous study indicated that the chemical compounds in cigarette smoke, including nicotine and polycyclic aromatic hydrocarbons, can alter the endocrine and central nervous systems and might subsequently affect the onset of menarche [21]. However, there have been only a few studies on this association [17,22,23]. To the best of our knowledge, there have been no studies showing a correlation of childhood passive smoking exposure with age at menarche in Asian women who have never smoked. The 2014 US Surgeon General (USSG) report on the health consequences of smoking did not include any studies on the association between passive smoking and age at menarche, although it included a study showing that maternal smoking during pregnancy was associated with earlier age at menarche [22,24]. We examined the associations between childhood passive smoke exposure and age at menarche in southern Chinese women who had never smoked. Chinese women, particularly those in southern China, have a very low smoking prevalence and are particularly suitable for studying the effects of passive smoking or secondhand smoke exposure.

## Design and Methods

### Guangzhou Biobank Cohort Study

We analyzed the baseline cross-sectional data from the Guangzhou Biobank Cohort Study (GBCS), which was conducted by the Guangzhou No. 12 Hospital and the Universities of Hong Kong and Birmingham. The GBCS is a continuing prospective cohort study of Guangzhou citizens aged 50 years or older that examines the environmental and genetic determinants of chronic diseases. Details of the methods have been reported elsewhere [25]. Briefly, the participants were recruited from "Guangzhou Health and Happiness Association for Respectable Elders", a community social and welfare association unofficially associated with the municipal government. It has more than 150 branches and about 100,000 members. These branches are distributed widely over the 12 districts of Guangzhou. The numbers of the branches and their members depend on the size of the district. Participants were randomly recruited from the association’s membership list [25]). Those receiving treatment for life-threatening diseases, such as cancer, were excluded. A detailed computer-based, standardized questionnaire was administered face-to-face by trained interviewers to collect information on age at menarche and various exposures, including a detailed assessment of childhood passive smoking exposure and personal disease history. The study recruited 30,518 participants (22,067 female, 72.3%), including 10,413 for phase 1 (September 2003 to November 2004), 10,017 for phase 2 (April 2005 to May 2006), and 10,088 for phase 3 (September 2006 to January 2008). Information on childhood family economic status, childhood family social status, frequency of eating meat and hunger as a child was only collected in phase 3.

### Measures

A person who had never smoked was defined as one who had smoked fewer than 100 cigarettes in his or her lifetime [26]. Childhood passive smoking exposure was defined as living with one or more smokers and being exposed to the smoker’s tobacco smoke for at least 15 minutes daily on more than one day every week in the same household [24] during childhood. Two self-reported measurements were used. The first on density was defined by the presence of zero, one, or two or more smokers living in the same household when the participant was a child. The other on frequency was defined as no exposure, < 5 days/week, or ≥5 days/week of exposure (one day was defined as exposure >15 min). Related questions were asked as follows: (1) Were there any smokers who lived with you in the same household during your childhood? 1. No, there were not; 2. Yes, there were; (2) If yes, how many smokers lived with you during childhood? 1. None; 2. One; 3. Two or more; (3) Did the smoker ever smoke in front you for more than 15 min in one day? 1. Yes; 2. No; and (4) What was the frequency of smokers smoking in front of you? 1. Never; 2. <5 days/week; 3. ≥5 days/week (one day was defined as an exposure >15 min). We used questions (1) and (3) to define passive smoking status in childhood; question (2) was used to define the density of passive smoking; and question (4) for frequency of passive smoking during childhood.

Age at menarche was self-reported. Related questions were asked to avoid recall errors in the age at menarche (What's your age when you had your first menstruation? What grade were you in at that time?). A total of 780 women who were ever smokers (current or former) were excluded. Among the 21,287 enrolled never-smoking women, 1,226 (5.8%) were excluded because of missing data on age at menarche.

We used the question "When you were a child, did your parents have a bicycle? A sewing machine? A watch?" to calculate the poverty index on childhood family economic status. Each "yes" to the three questions was scored one point and the total score was used as the poverty index (score 0–3). "Could your father read or write" was used to indicate childhood family social status. The following questions were used to reflect childhood nutrition status: (1) What was your frequency of meat eating as a child? 1. Never; 2. Once per year; 3. Once per month; 4. Once per week; 5. Once per day; and (2) What was your frequency of hunger as a child? 1. Never; 2. Once per year; 3. Once per month; 4. Once per week; 5. Once per day. These variables were only available for the phase 3 participants and were reported in details elsewhere [27].

### Statistical analysis

We used SPSS software, version 19.0 (Chicago, IL, USA), for data analysis. The significance level was set at a two-tailed P value of less than 0.05. We used linear regression models to examine the unadjusted and adjusted associations between childhood passive smoking exposure and age at menarche. Models A to C included all participants in the three phases: Model A was a crude model, Model B was adjusted for age, and Model C adjusted for age and education of the participants. Model D included only phase 3 data and was adjusted for age, education, childhood family economic status, childhood family social status, and frequency of eating meat and hunger as a child. Logistic regression models were used to calculate odds ratios (ORs) with 95% confidence intervals (95% CIs) for the unadjusted and adjusted associations between childhood passive smoking exposure and early age at menarche (defined as ≤13 years, as the age of 13 was the first quartile; vs. >13 years). The four logistic regression models followed the methods of linear regression above.

### Ethical considerations

The GBCS was approved by the Medical Ethics Committee of the Guangzhou Medical Association. All participants provided written informed consent before joining the study.

## Results

Of 20,903 women who had never smoked, 20,061 with all of the information available were included in the main analysis. The phase 3 participants (n = 6,679) were used for sensitivity analysis. The mean age was 60.9 years (range 50–95). Table 1 shows that 11,379 (56.7%) participants were exposed to passive smoking during childhood. The average age at menarche was 15 years (range 8–27). Nearly half of the participants had menarche when they were younger than 15 years (8,661, 43.2%).

**Table 1:** Baseline characteristics of female participants who had never smoked.

	Total (n = 20,061)	Childhood exposure (n = 11,379)	No childhood exposure (n = 8,682)
Age (years)			
50–59	10,281 (51.2)	6,666 (58.6)	3,615 (41.6)
60–69	7,338 (36.6)	3,696 (32.5)	3,642 (41.9)
≥70	2,442 (12.2)	1,017 (8.9)	1,425 (16.4)
Education			
≤Primary school	9,376 (46.7)	5,097 (44.8)	4,279 (49.3)
Middle school	9,474 (47.2)	5,655 (49.7)	3,819 (44.0)
≥College	1,211 (6.0)	627 (5.5)	584 (6.7)
Childhood home passive smoking exposure			
Number of smokers at home			
None	8,682 (43.3)	—	8,682 (100.0)
One	8,717 (43.5)	8,717 (76.6)	—
Two or more	2,662 (13.3)	2,662 (23.4)	—
Frequency of exposure			
None	8,682 (43.3)	—	8,682 (100.0)
<5 days/week	2,845 (14.2)	2,845 (25.0)	—
≥5 days/week	8,534 (42.5)	8,534 (75.0)	—
Age at menarche ≤13 years			
No	14,998 (74.8)	8,264 (72.6)	6,734 (77.6)
Yes	5,063 (25.2)	3,115 (27.4)	1,948 (22.4)

Data are n (%) for categorical values.

When age at menarche was analyzed as a continuous outcome, Model A to C showed that decreased average age at menarche was associated with increased number of smokers and increased frequency of exposure in the childhood household (all P for trend <0.001, Table 2). Model C shows that after adjusting for age and education, those who were exposed to more than two smokers had menarche 0.38 year (95% CI: 0.29–0.47) earlier, and those exposed for ≥5days/week, 0.19 year (95% CI: 0.13–0.25) earlier. Among the phase 3 participants (n = 6,679) (Model D), the results were similar.

**Table 2:** Linear regression between childhood passive smoking exposure and age at menarche (continuous) in women who had never smoked.

				95% CI			
		Mean (SD)	ß	Lower	Upper	Standard ß	P for trend
Number of smokers	None (reference)	15.3 (2.1)	Model A				<0.001
(n = 20,061)	1 smoker	15.0 (2.1)	-0.30	-0.36	-0.24	-0.07	
	≥2 smokers	14.8 (2.0)	-0.52	-0.61	-0.43	-0.09	
	None (reference)		Model B				<0.001
	1 smoker		-0.12	-0.18	-0.06	-0.03	
	≥2 smokers		-0.33	-0.42	-0.24	-0.05	
	None (reference)		Model C				<0.001
	1 smoker		-0.14	-0.20	-0.08	-0.03	
	≥2 smokers		-0.38	-0.47	-0.29	-0.06	
Frequency of exposure	None (reference)	15.3 (2.1)	Model A				<0.001
(n = 20,061)	<5 days/week	14.9 (2.0)	-0.40	-0.49	-0.31	-0.07	
	≥5 days/week	14.9 (2.1)	-0.33	-0.40	-0.27	-0.08	
	None (reference)		Model B				<0.001
	<5 days/week		-0.18	-0.27	-0.09	-0.03	
	≥5 days/week		-0.16	-0.22	-0.10	-0.04	
	None (reference)		Model C				<0.001
	<5 days/week		-0.20	-0.29	-0.12	-0.03	
	≥5 days/week		-0.19	-0.25	-0.13	-0.05	
Number of smokers	None (reference)		Model D				<0.001
(n = 6,679)	1 smoker		-0.10	-0.20	0.01	-0.02	
	≥2 smokers		-0.37	-0.52	-0.22	-0.06	
Frequency of exposure	None (reference)		Model D				0.008
(n = 6,679)	<5 days/week		-0.20	-0.34	-0.05	-0.03	
	≥5 days/week		-0.15	-0.25	-0.04	-0.03	

Model A: Unadjusted (n = 20,061). Model B: Adjusted for age (n = 20,061). Model C: Adjusted for age and education of the participants (n = 20,061). Model D: Adjusted for age and education, childhood family economic status, childhood family social status, frequency of eating meat and hunger as a child (phase 3 only; n = 6,697). The number of smokers and the frequency of exposure were included in the model separately.

Table 3 shows that the ORs of earlier age at menarche (≤13 years) increased with increasing number of smokers and frequency of exposure in the childhood household in Model A to C (all P for trend <0.001). The adjusted OR for ≥2 smokers and ≥5 days/week were 1.34 (95% CI: 1.21–1.48) and 1.17 (95% CI: 1.09–1.26), respectively. The ORs in Model D (phase 3) were smaller.

**Table 3:** Odds ratio (95% CI) of early age at menarche (≤13 years old) for childhood passive smoking exposure in women who have never smoked.

		N (%)	Model A OR (95% CI)	Model B OR (95% CI)	Model C OR (95% CI)	Model D OR (95% CI)
Age at menarche ≤13 years						
Number of smokers	None (reference)	1,948 (22.4)	1	1	1	1
	1 smoker	2,318 (26.6)	1.25 (1.17–1.34)	1.09 (1.01–1.17)	1.11 (1.03–1.19)	1.10 (0.97–1.24)
	≥2 smokers	797 (29.9)	1.48 (1.34–1.63)	1.28 (1.16–1.41)	1.34 (1.21–1.48)	1.26 (1.06–1.50)
	P for trend		<0.001	<0.001	<0.001	0.009
Frequency of exposure	None (reference)	1,948 (22.4)	1	1	1	1
	<5 days/week	790 (27.8)	1.33 (1.21–1.46)	1.12 (1.01–1.23)	1.14 (1.04–1.26)	1.19 (1.01–1.41)
	≥5 days/week	2,325 (27.2)	1.29 (1.21–1.39)	1.13 (1.06–1.22)	1.17 (1.09–1.26)	1.11 (0.98–1.26)
	P for trend		<0.001	0.001	<0.001	0.113

Model A: Unadjusted (n = 20,061). Model B: Adjusted for age (n = 20,061). Model C: Adjusted for age and education of the participants (n = 20,061). Model D: Adjusted for age and education, childhood family economic status, childhood family social status, frequency of eating meat and hunger as a child (phase 3 only; n = 6,697). The number of smokers and the frequency of exposure were included in the model separately.

The participants in the three phases were not very similar in terms of age, educational level, childhood passive smoking exposure, or age at menarche (S1 Table). Repeated analyses for each of the 3 phases separately showed similar results (S2–S5 Tables).

## Discussion

To the best of our knowledge, our study was the first to observe an association between childhood passive smoking and earlier age at menarche with a dose response relationship in Asia women who had never smoked.

Our study had several strengths. The sample was from southern China, where female smoking prevalence is low, and therefore, misclassification of smokers as never smokers was unlikely. Recall errors regarding age at menarche should be small. Consistent results were obtained after adjusting for age and education in a large sample and in sensitivity analysis on a subsample. The latter adjusted for childhood household socioeconomic and nutrition status which might be associated with age at menarche [18,28].

Previous research has suggested that exposure to secondhand cigarette smoke can have complex associations with reproductive function, mostly about the influence of prenatal tobacco smoke (PTS) on age at menarche, but the results have been mixed. A study in the U.S. did not find an association between PTS and age at menarche. However, the results might have been limited by the small sample size [23]. Our results were consistent with a California birth cohort study [22]. That study showed that girls with both high prenatal and high childhood passive smoke exposure had an adjusted mean age at menarche about 4 months younger than those unexposed. The New York City site data of the National Collaborative Perinatal Project (NCPP) showed a conflicting result, with heavy PTS and childhood passive smoking exposure associated with older age at menarche, whereas girls exposed to only childhood passive smoking were older at menarche (>12 years old vs. ≤12 years old), with an OR of 2.1 (95% CI: 1.0–4.3) [17]. However, most of their participants were of either European or African descent. Whether the differences between previous results and our results could be due to differences in ethnicity, cigarette smoke or other exposures should be studied further.

The criteria for early age at menarche vary with different regions, ethnicity and era, and there is a clear secular trend towards earlier age at menarche in Chinese women [29]. A previous study [17] used the median age at menarche of the participants as the cut-off point for early age at menarche. We used the first quartile (13 years) which should be more appropriate, and the results were similar to those with age at menarche as a continuous variable.

The mechanism of the association was not clear. However, previous studies have shown that passive smoking could alter endocrine and menstrual function in the female body [21,30], and maternal smoking exposure could affect embryo growth in the uterus [31]. Additionally, children's lungs and immune system are immature, making them more vulnerable to the adverse effects of secondhand smoke.

Moreover, the USSG 2014 report concluded that there was a suggestive causal relationship between passive smoking and breast cancer [24]. Our result that passive smoking could lead to early menarche can provide some preliminary supportive evidence because early menarche is a confirmed risk factor for breast cancer [4,5].

The major limitation of our study was the potential information bias, as the exposure and outcome data were all self-reported. A previous study showed that very few Chinese people believed that passive smoking exposure could affect their health [32], so bias from the awareness of a suspected or known association should have been minimal. Many previous studies [33] have shown fairly good validity of the questionnaires used to measure passive smoking exposure. Our previous report on the association between passive smoking and aortic arch calcification confirmed that the passive smoking data were robust [34]. The representativeness of our sample might be limited because the participants were all from an association concerned with health and welfare, but it is open to all older residents (≥50 years old) in Guangzhou for a small annual fee. Our participants might have been healthier than the general population. Finally, we did not have information about maternal smoking exposure, although maternal smoking by Chinese women during pregnancy was almost unheard of more than 50 years ago.

In conclusion, this study showed that childhood passive smoking was a risk factor for early age at menarche. The association, if causal, further supports the urgent need to promote smoking cessation in families with children, particularly young girls. The results would also support causal inference on the effects of childhood passive smoking on future adverse effects mediated by an early age of menarche.

## Supporting Information

S1 Dataset Dataset of 20061 participants. (XLS)

S1 Table Comparison of baseline data among women who have never smoked for 3 phases of participants. Data are n (%) for category values. Data are n (%) for category values.
(DOC)

S2 Table Relationship between childhood passive smoking exposure and age at menarche in women who have never smoked for 3 phases of participants. Model A: Unadjusted. Model B: Adjusted for the age and education of the participants.
(DOC)

S3 Table Odds ratio (95% CI) of early age at menarche (≤13 years) for childhood passive smoking exposure in phase 1 participants (n = 6,695). Model A: Unadjusted. Model B: Adjusted for the age and education of the participants.
(DOC)

S4 Table Odds ratio (95% CI) of early age at menarche (≤13 years) for childhood passive smoking exposure in phase 2 participants (n = 6,687). Model A: Unadjusted. Model B: Adjusted for the age and education of the participants.
(DOC)

S5 Table Odds ratio (95% CI) of early age at menarche (≤13 years) for childhood passive smoking exposure in phase 3 participants (n = 6,679). Model A: Unadjusted. Model B: Adjusted for the age and education of the participants.
(DOC)
