# Supplementary material for: Childhood Passive Smoking Exposure and Age at Menarche in Chinese Women Who Had Never Smoked: The Guangzhou Biobank Cohort Study
Source: PLoS One. 2015 Jul 17;10(7):e0130429. doi: 10.1371/journal.pone.0130429 (PMC4506068; doi:10.1371/journal.pone.0130429)
Supplement: S1 Table — Data are n (%) for category values. Data are n (%) for category values. (DOC) [file pone.0130429.s002.doc]

**S1 Table Comparison of baseline data among female never smokers of the 3 phases**

|  | N (%) in Phase 1 (n=6,695) | N (%) in Phase 2 (n=6,687) | N (%) in Phase 3(n=6,679) |
| --- | --- | --- | --- |
| Age (years) |  |  |  |
| 50-59 | 2,193(31.9) | 3,968 (59.3) | 4,174 (62.5) |
| 60-69 | 3,473 (51.9) | 2,118 (31.7) | 1,747 (26.2) |
| ≥70 | 2,083 (16.2) | 601 (9.0) | 758 (11.3) |
| Education |  |  |  |
| ≦Primary school | 3,737 (55.8) | 2,982 (44.6) | 2,657 (39.8) |
| Middle school | 2,563 (38.3) | 3,311 (49.5) | 3,600 (53.9) |
| ≥College school | 395 (5.9) | 394 (5.9) | 422 (6.3) |
| Childhood home exposure |  |  |  |
| None | 3,173 (47.4) | 2,838 (42.4) | 2,671 (40.0) |
| Yes | 3,522 (52.6) | 3,849 (57.6) | 4,008 (60.0) |
| Childhood home exposure |  |  |  |
| Number of smokers at home |  |  |  |
| None | 3,173 (47.4) | 2,838 (42.4) | 2,671 (40.0) |
| One | 2,638 (39.4) | 2,985 (44.6) | 3,094 (46.3) |
| Two or more | 884 (13.2) | 864 (12.9) | 914 (13.7) |
| Frequency of exposure |  |  |  |
| None | 3,173 (47.4) | 2,838 (42.4) | 2,671 (40.0) |
| <5 days/week | 289 (4.3) | 1,544 (23.1) | 1,012 (15.2) |
| ≥5 days/week | 3,233 (48.3) | 2,305 (34.5) | 2,996 (44.9) |
| Age at menarche ≤13 years |  |  |  |
| No | 5,274 (78.8) | 4,917 (73.5) | 4,807 (72.0) |
| Yes | 1,421 (21.2) | 1,770 (26.5) | 1,872 (28.0) |

*Data are n (%) for category value*
