# Supplementary material for: Childhood Passive Smoking Exposure and Age at Menarche in Chinese Women Who Had Never Smoked: The Guangzhou Biobank Cohort Study
Source: PLoS One. 2015 Jul 17;10(7):e0130429. doi: 10.1371/journal.pone.0130429 (PMC4506068; doi:10.1371/journal.pone.0130429)
Supplement: S2 Table — Model A: Unadjusted. Model B: Adjusted for the age and education of the participants. (DOC) [file pone.0130429.s003.doc]

**S2 Table Relation between childhood passive smoking exposure and age at menarche in female never smokers of the 3 phases**

|  |  |  |  | 95%CI | |  |  |
| --- | --- | --- | --- | --- | --- | --- | --- |
|  |  | Mean (SD) | ß | lower | upper | Standard ß | P value |
| Phase 1 (n=6,695) |  |  |  |  |  |  |  |
| Number of smokers | None (reference) | 15.5 (2.1) | Model A |  |  |  | <0.001 |
|  | 1 smoker | 15.3 (2.0) | -0.26 | -0.37 | -0.15 | -0.06 |  |
|  | ≥2 smokers | 15.1 (2.1) | -0.50 | -0.65 | -0.34 | -0.08 |  |
|  | None (reference) |  | Model B |  |  |  | <0.001 |
|  | 1 smoker |  | -0.20 | -0.30 | -0.10 | -0.05 |  |
|  | ≥2 smokers |  | -0.45 | -0.60 | -0.30 | -0.07 |  |
| Frequency of exposure | None (reference) | 15.5 (2.1) | Model A |  |  |  | <0.001 |
|  | <5 days/week | 15.2 (1.9) | -0.27 | -0.52 | 0.20 | -0.03 |  |
|  | ≥5 days/week | 15.2 (2.1) | -0.32 | -0.43 | 0.22 | 0.08 |  |
|  | None (reference) |  | Model B |  |  |  | <0.001 |
|  | <5 days/week |  | -0.20 | -0.44 | 0.04 | -0.02 |  |
|  | ≥5 days/week |  | -0.27 | -0.37 | -0.17 | -0.06 |  |
| Phase 2 (n=6,687) |  |  |  |  |  |  |  |
| Number of smokers | None (reference) | 15.1 (2.0) | Model A |  |  |  | <0.001 |
|  | 1 smoker | 14.8 (2.0) | -0.25 | -0.35 | -0.15 | -0.06 |  |
|  | ≥2 smokers | 14.7 (1.9) | -0.40 | -0.55 | -0.25 | -0.07 |  |
|  | None (reference) |  | Model B |  |  |  | <0.001 |
|  | 1 smoker |  | -0.10 | -0.20 | -0.01 | -0.03 |  |
|  | ≥2 smokers |  | -0.26 | -0.41 | -0.12 | -0.04 |  |
| Frequency of exposure | None (reference) | 15.1 (2.0) | Model A |  |  |  | <0.001 |
|  | <5 days/week | 14.9 (2.0) | -0.25 | -0.37 | 0.12 | -0.05 |  |
|  | ≥5 days/week | 14.8 (2.0) | -0.31 | -0.42 | -0.20 | -0.07 |  |
|  | None (reference) |  | Model B |  |  |  | 0.007 |
|  | <5 days/week |  | -0.13 | -0.25 | -0.01 | -0.03 |  |
|  | ≥5 days/week |  | -0.15 | -0.25 | -0.04 | -0.03 |  |
| Phase 3 (n=6,679) |  |  |  |  |  |  |  |
| Number of smokers | None (reference) | 15.2 (2.2) | Model A |  |  |  | <0.001 |
|  | 1 smoker | 14.9 (2.1) | -0.32 | -0.43 | -0.21 | -0.07 |  |
|  | ≥2 smokers | 14.6 (2.0) | -0.63 | -0.79 | -0.47 | -0.10 |  |
|  | None (reference) |  | Model B |  |  |  | <0.001 |
|  | 1 smoker |  | -0.12 | -0.22 | -0.01 | -0.03 |  |
|  | ≥2 smokers |  | -0.42 | -0.57 | -0.27 | -0.07 |  |
| Frequency of exposure | None (reference) | 15.2 (2.2) | Model A |  |  |  | <0.001 |
|  | <5 days/week | 14.8 (2.1) | -0.38 | -0.54 | -0.23 | -0.06 |  |
|  | ≥5 days/week | 14.8 (2.1) | -0.39 | -0.50 | -0.28 | -0.09 |  |
|  | None (reference) |  | Model B |  |  |  | 0.001 |
|  | <5 days/week |  | -0.20 | -0.35 | -0.06 | -0.03 |  |
|  | ≥5 days/week |  | -0.18 | -0.29 | -0.07 | -0.04 |  |

*Model A: Unadjusted*

*Model B: Adjusted for age and education of the participants*
