# Supplementary material for: Childhood Passive Smoking Exposure and Age at Menarche in Chinese Women Who Had Never Smoked: The Guangzhou Biobank Cohort Study
Source: PLoS One. 2015 Jul 17;10(7):e0130429. doi: 10.1371/journal.pone.0130429 (PMC4506068; doi:10.1371/journal.pone.0130429)
Supplement: S3 Table — Model A: Unadjusted. Model B: Adjusted for the age and education of the participants. (DOC) [file pone.0130429.s004.doc]

**S3 Table Odds Ratio (95% CI) of early age at menarche (≤13 years) for childhood passive smoking exposure in phase 1 participants (n=6,695)**

|  |  | N (%) | Model A (95%CI) | Model B (95%CI) |
| --- | --- | --- | --- | --- |
| Age at menarche ≤13 years | |  |  |  |
| Number of smokers | None (reference) | 613 (19.3) | 1 | 1 |
|  | 1 smoker | 579 (21.9) | 1.17 (1.03-1.33) | 1.12 (0.98-1.27) |
|  | ≥2 smokers | 229 (25.9) | 1.46 (1.22-1.74) | 1.41 (1.18-1.69) |
|  | P |  | <0.001 | <0.001 |
| Frequency of exposure | None (reference) | 613 (19.3) | 1 | 1 |
|  | <5 days/week | 55 (19.0) | 0.98 (0.72-1.33) | 0.92 (0.67-1.26) |
|  | ≥5 days/week | 753 (23.3) | 1.27 (1.13-1.43) | 1.21 (1.07-1.37) |
|  | P |  | <0.001 | 0.002 |

*Model A: Unadjusted*

*Model B: Adjusted for age and education of the participants*
