# Supplementary material for: Childhood Passive Smoking Exposure and Age at Menarche in Chinese Women Who Had Never Smoked: The Guangzhou Biobank Cohort Study
Source: PLoS One. 2015 Jul 17;10(7):e0130429. doi: 10.1371/journal.pone.0130429 (PMC4506068; doi:10.1371/journal.pone.0130429)
Supplement: S4 Table — Model A: Unadjusted. Model B: Adjusted for the age and education of the participants. (DOC) [file pone.0130429.s005.doc]

**S4 Table Odds Ratio (95% CI) of early age at menarche (≤13 years) for childhood passive smoking exposure in phase 2 participants (n=6,687)**

|  |  | N (%) | Model A (95%CI) | Model B (95%CI) |
| --- | --- | --- | --- | --- |
| Age at menarche ≤13 years | |  |  |  |
| Number of smokers | None (reference) | 674 (23.7) | 1 | 1 |
|  | 1 smoker | 829 (27.8) | 1.24 (1.08-1.33) | 1.10 (0.98-1.25) |
|  | ≥2 smokers | 267 (30.9) | 1.44 (1.21-1.70) | 1.30 (1.09-1.54) |
|  | P |  | <0.001 | 0.003 |
| Frequency of exposure | None (reference) | 674 (23.7) | 1 | 1 |
|  | <5 days/week | 426 (27.6) | 1.22(1.06-1.41) | 1.12 (0.97-1.30) |
|  | ≥5 days/week | 670 (29.1) | 1.32(1.16-1.49) | 1.16 (1.02-1.32) |
|  | P |  | <0.001 | 0.022 |

*Model A: Unadjusted*

*Model B: Adjusted for age and education of the participants*
