# Supplementary material for: Childhood Passive Smoking Exposure and Age at Menarche in Chinese Women Who Had Never Smoked: The Guangzhou Biobank Cohort Study
Source: PLoS One. 2015 Jul 17;10(7):e0130429. doi: 10.1371/journal.pone.0130429 (PMC4506068; doi:10.1371/journal.pone.0130429)
Supplement: S5 Table — Model A: Unadjusted. Model B: Adjusted for the age and education of the participants. (DOC) [file pone.0130429.s006.doc]

**S5 Table Odds Ratio (95% CI) of early age at menarche (≤13 years) for childhood passive smoking exposure in phase 3 participants (n=6,679)**

|  |  | N (%) | Model A (95%CI) | Model B (95%CI) |
| --- | --- | --- | --- | --- |
| Age at menarche ≤13 years | |  |  |  |
| Number of smokers | None (reference) | 661 (24.7) | 1 | 1 |
|  | 1 smoker | 910 (29.4) | 1.27 (1.13-1.42) | 1.11 (0.98-1.25) |
|  | ≥2 smokers | 301 (32.9) | 1.49 (1.27-1.76) | 1.32 (1.12-1.57) |
|  | P |  | <0.001 | 0.002 |
| Frequency of exposure | None (reference) | 661 (24.7) | 1 | 1 |
|  | <5 days/week | 309 (30.5) | 1.34 (1.14-1.57) | 1.19 (1.01-1.41) |
|  | ≥5 days/week | 902 (30.1) | 1.31 (1.17-1.47) | 1.14(1.01-1.29) |
|  | P |  | <0.001 | 0.041 |

*Model A: Unadjusted*

*Model B: Adjusted for age and education of the participants*
